# Amiodarone Induces Overexpression of *Similar to Versican b* to Repress the EGFR/Gsk3b/Snail Signaling Axis during Cardiac Valve Formation of Zebrafish Embryos

**DOI:** 10.1371/journal.pone.0144751

**Published:** 2015-12-09

**Authors:** Hung-Chieh Lee, Hao-Chan Lo, Dao-Ming Lo, Mai-Yan Su, Jia-Rung Hu, Chin-Chieh Wu, Sheng-Nan Chang, Ming-Shen Dai, Chia‐Ti Tsai, Huai-Jen Tsai

**Affiliations:** 1 Institute of Biomedical Sciences, Mackay Medical College, New Taipei City, Taiwan; 2 Institute of Molecular and Cellular Biology, National Taiwan University, Taipei, Taiwan; 3 Division of Hematology/Oncology, Tri-Service General Hospital, National Defense Medical Centre, Taipei, Taiwan; 4 Cardiovascular Center, National Taiwan University Hospital Yun Lin Branch, Yun Lin, Taiwan; 5 Division of Cardiology, Department of Internal Medicine, National Taiwan University College of Medicine and Hospital, Taipei, Taiwan; Northwestern University, UNITED STATES

## Abstract

Although Amiodarone, a class III antiarrhythmic drug, inhibits zebrafish cardiac valve formation, the detailed molecular pathway is still unclear. Here, we proved that Amiodarone acts as an upstream regulator, stimulating *similar to versican b (s-vcanb)* overexpression at zebrafish embryonic heart and promoting *cdh-5* overexpression by inhibiting *snail1b* at atrioventricular canal (AVC), thus blocking invagination of endocardial cells and, as a result, preventing the formation of cardiac valves. A closer investigation showed that an intricate set of signaling events ultimately caused the up-regulation of *cdh5*. In particular, we investigated the role of EGFR signaling and the activity of Gsk3b. It was found that knockdown of EGFR signaling resulted in phenotypes similar to those of Amiodarone-treated embryos. Since the reduced phosphorylation of EGFR was rescued by knockdown of *s-vcanb*, it was concluded that the inhibition of EGFR activity by Amiodarone is *s-vcanb*-dependent. Moreover, the activity of Gsk3b, a downstream effector of EGFR, was greatly increased in both Amiodarone-treated embryos and EGFR-inhibited embryos. Therefore, it was concluded that reduced EGFR signaling induced by Amiodarone treatment results in the inhibition of Snail functions through increased Gsk3b activity, which, in turn, reduces snail1b expression, leading to the up-regulation the *cdh5* at the AVC, finally resulting in defective formation of valves. This signaling cascade implicates the EGFR/Gsk3b/Snail axis as the molecular basis for the inhibition of cardiac valve formation by Amiodarone.

## Introduction

Cardiac valves play crucial roles in heart function by maintaining unidirectional blood flow in the heart. Cardiac valves contain a heterogeneous population of valvular endothelial cells (VECs) and valvular interstitial cells (VICs), which maintain valve homeostasis and structural leaflet integrity. Within the developing and mature value, VICs are reported to mediate the processes and maintain the structure of the tri-laminar extracellular matrix (ECM) throughout life [1, 2]. In mice, chick and human, the formation of cardiac valves is restricted to the atrioventricular canal (AVC) and outflow tract regions of the looping heart. The endocardial cushions, which are the primordia of the cardiac valves and septae, are formed in these regions by tissue patterning and epithelial-mesenchymal transition (EMT) known for its involvement in cell differentiation and developmental processes, but also its initiation of cancer metastasis [3]. These mesenchymal precursors migrate into the cardiac jelly and proliferate, thereby expanding the endocardial cushion via a series of signaling pathways [4–10]. The extracellular matrix (ECM) in heart valves provides both physical support for cellular growth and biologically active structure for many cellular functions [11]. These signaling pathways exhibit dynamic temporal regulation throughout development to form the final three-dimensional valve structure. However, the signaling pathways involved in this bioprocess are not completely understood.

ECM is a highly organized three-dimensional structure that plays many physiological and pathological roles. ECM proteins are necessary for AVC differentiation [12]. In particular, the ECM of heart valves is stratified into elastin-, proteoglycan- and collagen-rich layers that confer distinct biomechanical properties to the valve leaflets and supporting apparatus of heart. Versican, an important ECM component, is a large chondroitin sulfate proteoglycan belonging to the lectican family [13]. Alternative splicing of mammalian *versican* mRNA generates at least four isoforms, termed V0, V1, V2, and V3, each having different functions. For example, the V2 isoform inhibits axonal and neurite growth [14], but the V1 isoform promotes neurite outgrowth [15]. Sheng et al. [16] also reported that the V1 isoform is able to enhance cell proliferation, while the V2 isoform inhibits cell proliferation. These opposing effects suggest a dynamically balanced expression pattern between these two isoforms that could provide a suitable extracellular environment for normal proliferation and survival of cells. Moreover, each Versican isoform may play its own role during cardiac valve formation. In the heart, total absence of the *versican* gene halts heart development at a stage prior to the heart's pulmonary/aortic outlet segment growth and causes early embryonic lethality [17]. It has been reported that the *Vcan* ^*(tm1Zim)*^ mutant, which is unable to express the V0 and V2 forms of Versican, can survive until adulthood; however, it displays defects with smaller cushions/valve leaflets [18]. While interesting, these findings have not begun to identify the role(s) of each Versican isoform during cardiac development.

Cardiac valve development is characterized by morphogenetic complexity, and, as such, it is very sensitive to teratogenic factors. Abnormal developmental of cardiac valves causes congenital heart disease. Blue et al. [19] reported the frequency of congenital valve malformations as high as 5% of live births, and 80% of them were attributed to an unknown etiology, indicating that our understanding of the molecular events governing the complex processes of cardiac endocardial cushion formation and valvulogenesis is limited.

Zebrafish is a genetically tractable model that offers unique advantages for *in vivo* studies, especially for studying heart development. The zebrafish heart can be observed under a dissecting microscope soon after formation of the primitive heart tube. The functional valves are formed by 48 hours post-fertilization (hpf), and they are complete by approximately 55 hpf. The AVC in zebrafish is molecularly defined by expression of *versican*, *bmp4* and *notch1b* [20]. The endocardial cushions are remodeled into the mature valve leaflets which require Notch [21, 22], NFAT [23], Wnt [24] and ErbB [25] signaling, as reported in mouse, suggesting that the major cellular and molecular events of cardiac valve development in zebrafish are largely conserved to amniotes.

Amiodarone is an effective and widely used anti-arrhythmic drug. Cardiac arrhythmia is also common in pregnant women, and the toxic effect of Amiodarone on embryogenesis has been controversial and is an important issue. We previously reported that Amiodarone inhibits zebrafish cardiac valve formation by inducing the expression of *versican* and chromodomain-helicase-DNA-binding protein 5 tumor suppressor gene (*cdh5*; VE-cadherin) at the AVC of zebrafish embryos [26]. Since *cdh5* can be overexpressed by Amiodarone, this finding suggested that Amiodarone may interfere with EMT processes during cardiogenesis. However, compared to mammalian Versican, zebrafish Versican is largely unknown, except for its restriction to the AVC endocardium by 48 hpf [24]. Furthermore, we do not know the molecular mechanism underlying Amiodarone’s inhibition of cardiac valve formation. Here, our results implicated the EGFR/Gsk3b/Snail signaling axis as the molecular basis for Amiodarone's inhibition of cardiac valve formation. In brief, Amiodarone induces ectopic expression of only one Versican isoform, *similar to versican b* (*s-vcanb*), which represses EGFR activity, thereby increasing Gsk3b activity, which, in turn, down-regulates the expression of *snail1b*, leading, finally, to the up-regulation of *cdh5* at the AVC and improper development of cardiac valves of zebrafish heart.

## Material and Methods

### Zebrafish husbandry, animal studies, and microscopy observation

Zebrafish AB strain was maintained in aquaria according to standard procedures described by Westerfield et al. [27]. The experiments and treatments of this animal have been reviewed and approved by the National Taiwan University Institutional Animal Care and Use Committee with ethics approval number NTU-101-EL-115. Fluorescence was visualized with a fluorescent stereomicroscope (MZ FLIII, Leica) and a confocal spectral microscope (TCS SP5, Leica).

### Drug treatment with zebrafish embryos

EGFR inhibitor AG 1478 (CalBiochem) was dissolved in DMSO and stocked as 1 mM at -20°C. The working concentration used in this study was 7 μM. Amiodarone (Sigma) was dissolved in water at 65°C for 2 h and stocked as 900 μM at 4°C. Before use, the solution was dissolved again at 65°C for 1 h. In the control group, 100 embryos were placed in a 9-cm dish filled with a volume of 30 ml embryo medium containing 0.2 mM 1-phenyl-2-thio-urea (Sigma). In the experimental group, the protocol was identical to the control group, except that embryos at different stages were treated with concentrations of Amiodarone that ranged from 3 to 30 μM, and embryos were exposed to treatment from 12 to 84 h. Long-term treatment was performed during 12–72 hpf, in which the specification stage of valve formation at 36–55 hpf and the invagination stage of valve formation at 55 hpf were included. Treatment during 12–48 hpf was used to examine the expressions of *versican* and *cdh5* genes. During treatment, Amiodarone was refreshed every 24 h, and after treatment, embryos were washed twice with embryo medium, collected into a new 9-cm dish, and then incubated at 28°C.

### Knockdown experiments with antisense morpholino oligomers (MO) and microinjection

To design the *vcana*-MO, a 500 bp *vcana* fragment containing a 250-bp 5’UTR and a 250-bp coding region was isolated by Reverse transcription-polymerase chain reaction (RT-PCR) and confirmed by sequencing. The following MOs were purchased from GeneTools (USA): *vcana*-MO (aMO)(AGGAAGATACCCATATTTCTGCTGA); *s-vcanb*-MO (vMO)(CTGAAACACC- CATGGGAGTGGACAT); *snail1b*-MO (TTGACAAGAAATGAGCGTGGCATCT) [28]; *cdh5-*MO (TTTACAAGACCGTCTCCTTTCCAA) [29]; *troponin T2a*, *cardiac*-MO (CATGTTTGCTCTGATCTGACACGCA) [30]; and standard control-MO (CCTCTTACCTCAGTTACAATTTATA). All MOs were prepared at a stock concentration of 1 mM and diluted to the desired concentration, specifically, 8, 12 and 16 ng for *vcana*-MO and *s-vcanb*-MO; 4, 8, 12 and 16 ng for control-MO; and 0.8, 1.2, 1.6 and 2 ng for *snail1b-*MO and *cdh5*-MO. The standard control-MO served as negative control. The microinjection procedures were previously described [26]. The specific of *vcana*-MO was confirmed (S1 Fig).

### Plasmid construction

The zebrafish *s-vcanb* contains a 5-kb coding region. Therefore, we used eight primers to amplify four fragments containing *s-vcanb*. The first 1.5 kb *s-vcanb* fragment was amplified by Z-s-vcanb *Cla*I F1 and Z-s-vcanb *Mfe*I R1; the 1.5 to 2 kb fragment was amplified by Z-s-vcanb *MfeI* F2 and Z-s-vcanb *Hpa*I R2; the 2–3.5 kb fragment was amplified by Z-s-vcanb *Hpa*I F3 and Z-s-vcanb *Xba*I R3; and the 3.5–5 kb fragment was amplified by Z-s-vcanb *Xba*I F4 and Z-s-vcanb *Afe*I R4. These four DNA fragments were ligated using their specific restriction enzymes’ cutting sites. Finally, the full length zebrafish *s-vcanb* was inserted into pCS2+ vector. The zS-vcanb-mE was previously described in Lee et al. [31]. To prove the specific effectiveness of *vcana*-MO, we designed a synthetic aMO-target-*eGFP* mRNA, in which the *vcana* cDNA, including *vcana*-MO target sequence, is fused in frame with *egfp* cDNA.

### Whole-mount *in situ* hybridization (WISH)

To examine the expression patterns of these three versican family genes, we designed specific probes to perform WISH. For example, the coding region 1.0–2.0 kb of *vcana* cDNA served as a *vcana* probe, and the coding region 3.3–3.9 kb of *vcanb* cDNA served as a *vcanb* probe, while the coding region 0.01–1.5 kb of *s-vcanb* served as *s-vcanb* probe. The full length coding sequence of *snail1a*, *snail1b*, *snail2*, and *snail3* were isolated by RT-PCR, inserted into plasmid pGEMTeasy (Promega) and confirmed by sequencing. After we cloned the partial DNA fragments of the desired genes, the probes were labeled by Digoxigenin (DIG). After permeabilization, embryos were hybridized overnight. Then, embryos were incubated with anti-DIG antibody (Roche; 1:8,000), stained and observed under a fluorescent stereomicroscope (MZ FLIII, Leica). Numbers shown in the bottom corner indicated number of embryos with similar staining patterns out of total number of embryos examined. All the primers used in this study were listed in S1 Table.

### Western blot analysis

The embryos were dechorionated and deyolked with two extra washing steps. Deyolked samples were dissolved in 2 μl of 2 X SDS sample buffer for each embryo and incubated for 5 min at 95°C. After full-speed centrifugation for 1 min in a microcentrifuge to remove insoluble particles, total proteins extracted from embryos were analyzed on a 12% SDS-PAGE gel, and Western blot analysis was performed using antiserum against Cdh5 (Santa Cruz; 1:1,000 dilution), EGFR (Millipore;1:1,000 dilution), pEGFR(Y845) (Cell Signaling; 1:1,000 dilution), Snail1b (Cell Signaling; 1:500 dilution), S-vcanb (Santa Cruz; 1:200 dilution), Gsk3b (BD; 1:1000 dilution), pGsk3b (Cell Signaling; 1:1,000 dilution), Versican V1 (Abcam; 1:1,000 dilution), Versican V2 (Thermo; 1:1,000 dilution), AKT (Cell Signaling; 1:1,000 dilution), pAKT (Cell Signaling; 1:1,000 dilution), ERK (Cell Signaling;1:1,000 dilution) and pERK (Cell Signaling; 1:1,000 dilution). Anti-α-tubulin and anti-β-actin served as a protein loading control. Quantitative ratio was presented as protein level that was normalized with that of internal control (such as α-tubulin or β-actin) using the NIH ImageJ software.

### Statistical analysis

Statistical analysis of the intensity of band shown on Western blot was averaged from three independent experiments, and presented as mean ± S.D., and difference levels were analyzed using Student’s *t*-test. **P<0.01 and *** *P*<0.005 indicated the levels of significant difference.

## Results

### Amiodarone causes overexpression of *s-vcanb* resulting in decreased *snail1b* and, consequently, increased *cdh5* in zebrafish embryos

**Fig 1:**
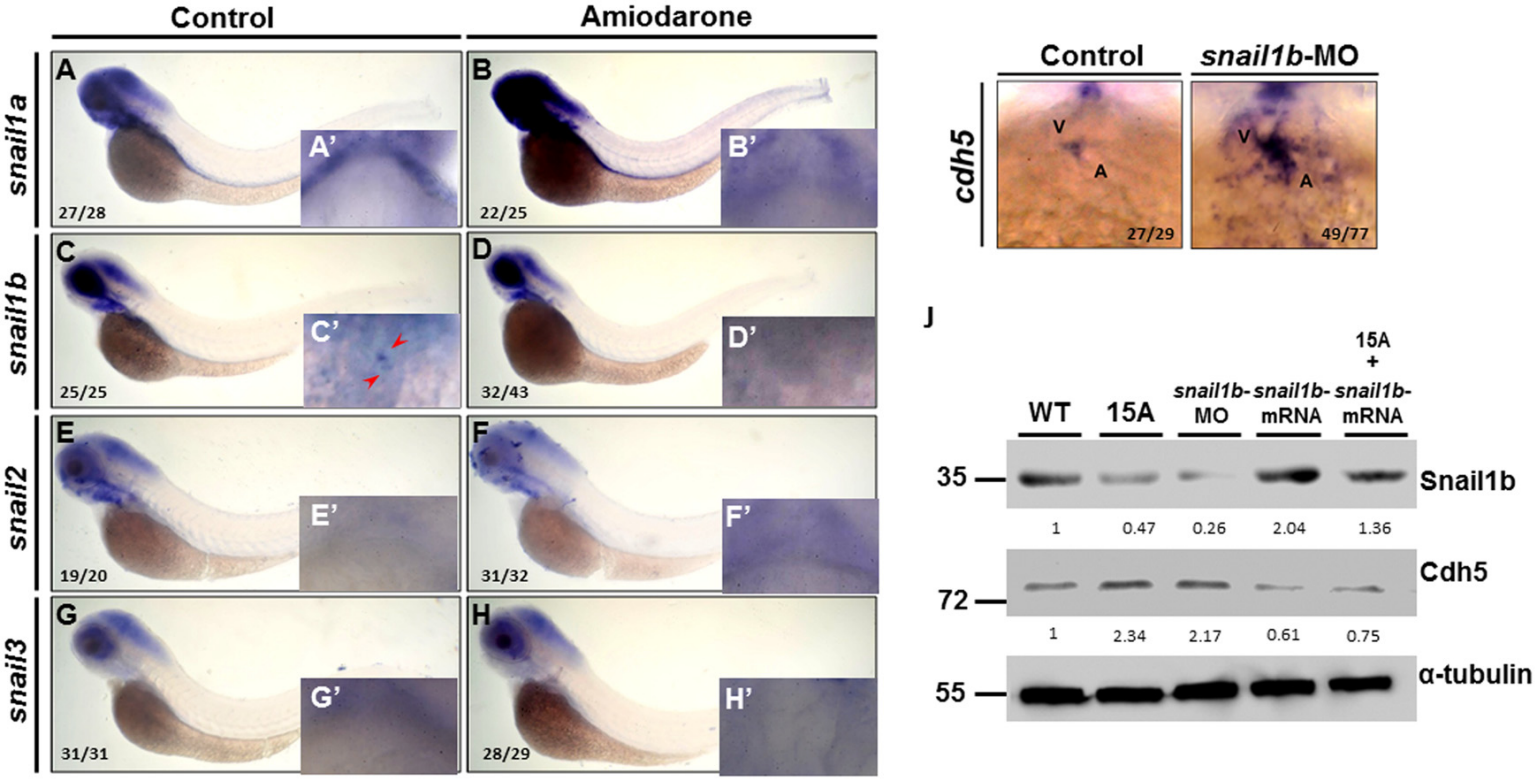
Amiodarone causes the ectopic expression of *cdh5* through its repression of *snail1b* expression. (A-H) WISH of *snail1a* (A, B) *snail1b* (C, D), *snail2* (E, F) and *snail3* (G, H) in indicated embryos during 72 hpf. Control embryos were treated with DMSO. 72 hpf zebrafish embryos treated with 15 μM Amiodarone from 55 to 72 hpf. (A-H) Lateral view of 72 hpf embryos. (A’ and H’) Ventral view of 72 hpf embryos which focus on the heart region. WISH revealed that *snail1a*, *snail2* and *snail3* were not detected at the AVC, while *snail1b* is detected at the AVC (C’ red arrow). Incubation of 15 μM Amiodarone from 55 to 72 hpf did not influence *snail1a* or *snail1b* at head region, but the expression of *snail1b* at AVC was lost (D’ red arrow). (I) WISH of *cdh5* in control-MO or *snail1b*-MO-injected embryos. (J) Western blot analysis of Cdh5 protein level in control-MO (Ctrl), Amiodarone-treated embryos (15A), *snail1b*-MO-injected embryos, *snail1b*-mRNA-injected embryos, and Amiodarone-treated plus *snail1b-*mRNA-injected embryos. V: ventricle, A: atrium. The number of embryos displaying similar pattern was indicated in each figure. The relative intensities of each protein were as indicated. The α-tubulin was used as an internal control.

Since Amiodarone can induce *cdh5* transcripts at the AVC [26], the Snail family of proteins, which functions as transcriptional repressors of the *cdh5* gene [32], may also be influenced by Amiodarone. To confirm this, four members of the Snail family of repressors were analyzed in zebrafish for their expression pattern using WISH. Only *snail1b* was expressed in the AVC (Fig 1C’). When embryos were treated with Amiodarone, *snail1b* transcripts were lost at the AVC (Fig 1D’). Meanwhile, knockdown of *snail1b* by specific MO increased *cdh5* expression at the AVC (Fig 1I). Western blot analysis also demonstrated that the protein level of Cdh5 was increased in the Amiodarone-treated embryos and the *snail1b*-MO-injected embryos (Fig 1J and S2A Fig), but reduced in *snail1b*-mRNA-injected embryos. Importantly, it was found that overexpression of *snail1b* in Amiodarone-treated embryos reduced the Cdh5 protein level (Fig 1J and S2A Fig), which was corresponding with that the reduced Cdh5 protein level was observed in the overexpression of *snail1b* alone in embryos. Thus, we suggested that Amiodarone causes the ectopic expression of *cdh5* through its repression of *snail1b* expression.

It has been reported that the ectopic expression of *cdh5* caused by Amiodarone treatment is, in fact, dependent on *versican* overexpression [26]. Moreover, as determined by the knockdown experiments above, the repressor *snail1b* is lost at the AVC, resulting in increasing *cdh5* expression. Therefore, it can be concluded that the effects of Amiodarone on *snail1b/cdh5* signaling are dependent on *versican* overexpression [26]. Based on the NCBI database, the Versican family genes in zebrafish include three isoforms: *vcana*, *vcanb* and *s-vcanb*. The *vcana* gene is located at chromosome 5 and usually serves as a paraxial mesoderm marker during early gastrulation [33] and an AVC marker during heart development [20, 24]. The *vcanb* gene, located at chromosome 10, plays roles on dermal bone development [34]. Cheng et al. [35] reported that HNF factor could induce *vcanb* to express in liver tissue. Many previous studies have focused on *vcana* and *vcanb* genes. However, the *s-vcanb* gene is understudied in the literature.

**Fig 2:**
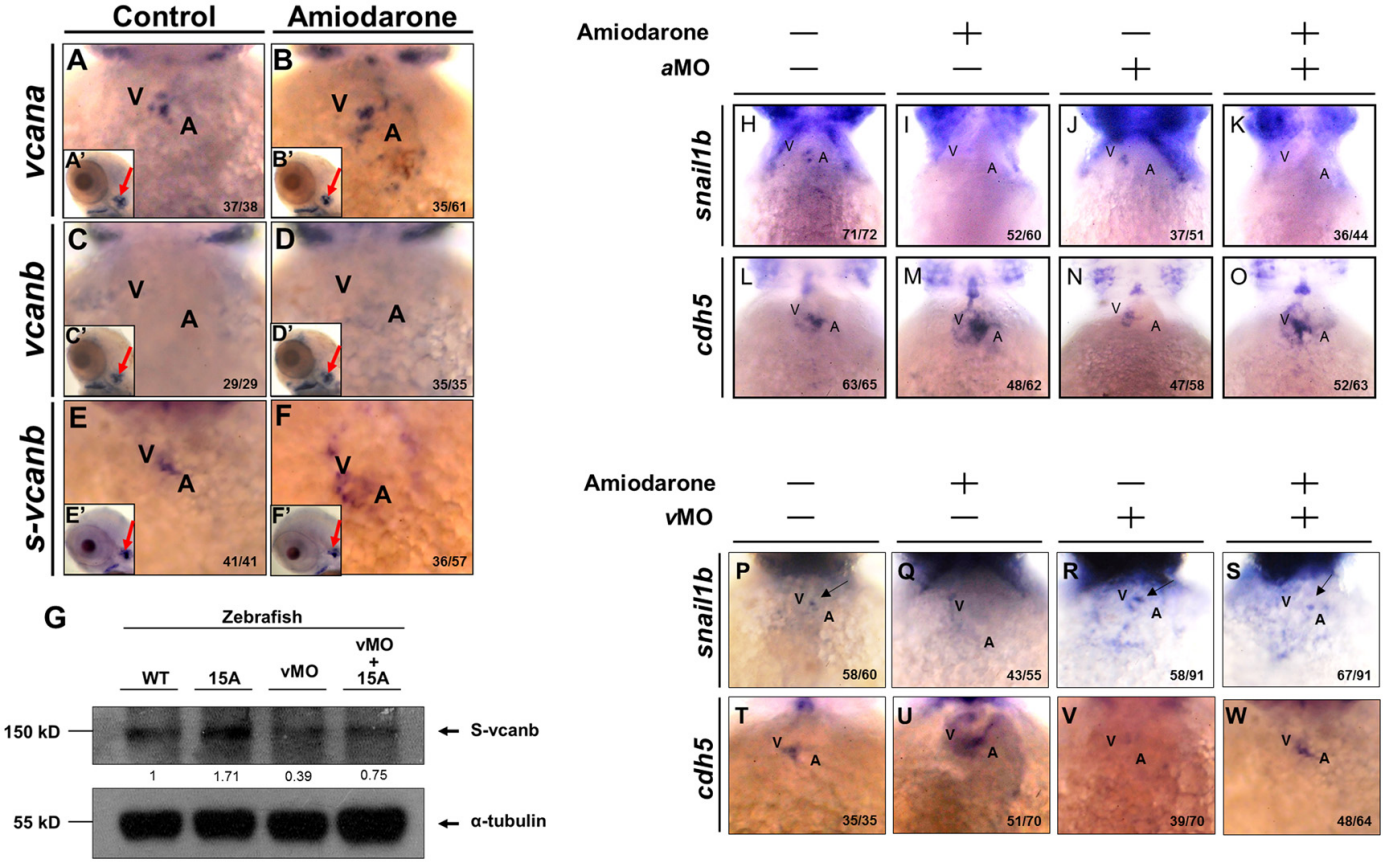
Reduced Snail1b and increased Cdh5 caused by Amiodarone treatment during valve formation are dependent on S-vcanb. Using WISH to detect the expression of *vcana* (A, B), vcanb (C, D) and *s-vcanb* (E, F) in embryos at 72 hpf was indicated. The head region of embryos treated with either DMSO (Control; A, C, E) or 15 μM Amiodarone (B, D, F) from 55 to 72 hpf. (A’-F’) indicates the head region of each embryo. Red arrows indicate the otic vesicle. The *vcana* and *s-vcanb* in the Amiodarone-treated embryos were highlighted. Their ectopic expression occurred only at the heart region. In other tissues, such as otic vesicle, no change was observed. (G) Western blot analysis of S-vcanb protein in wild-type embryos, embryos at 72 hpf after treatment with 15 μM Amiodarone from 55 to 72 hpf (15 A), embryos injected with *s-vcanb*-MO at one-cell stage (lane 3), and embryos injected with *s-vcanb*-MO at one-cell stage combined with 15 μM Amiodarone treatment from 55 to 72 hpf (lane 4). The α-tubulin gene served as the internal control. (H-W) Using WISH to detect the expressions of *snail1b* (H-K, P-S) and *cdh5* (L-O, T-W) in WT embryos (H, L, P, T), Amiodarone-treated embryos (I, M, Q, U), *vcana*-MO-injected embryos (J, N), *s-vcanb*-MO-injected embryos (R, V), Amiodarone-treatment combined with *vcana*-MO injection (K, O), and Amiodarone-treatment combined with *s-vcanb*-MO injection (S, W). V: ventricle, A: atrium. The number of embryos displaying a similar pattern was indicated in each figure.

To examine the expression patterns of these three versican family genes and to further determine which type of Versican is affected by Amiodarone, we performed WISH and found that only *vcana* (Fig 2A) and *s-vcanb* (Fig 2E) were expressed at the AVC during zebrafish cardiac development. Furthermore, Amiodarone-treated embryos displayed ectopic expression of *vcana* (Fig 2B) and *s-vcanb* (Fig 2F) at the AVC. The *vcanb* was not detected at the AVC during 72 hpf (Fig 2C), and its expression pattern remained unchanged after Amiodarone treatment (Fig 2D).

Interestingly, however, knockdown of *vcana* by injection of *vcana-*specific MO (aMO) did not alter the expression of *snail1b* (Fig 2J *vs*. 2H) or *cdh5* (Fig 2N *vs*. 2L), whereas *s-vcanb-*MO (vMO)-injected embryos exhibited increased expression of *snail1b* (Fig 2R *vs*. 2P), but greatly reduced *cdh5* expression (Fig 2V *vs*. 2T). Additionally, knockdown of *vcana* in Amiodarone-treated embryos did not rescue the expression patterns of *snail1b* (Fig 2K *vs*. 2H) or *cdh5* (Fig 2O *vs*. 2L) at the AVC, whereas knockdown of *s-vcanb* blocked the effects of Amiodarone because *snail1b* was still present (Fig 2S *vs*. 2P), and *cdh5* was not ectopically expressed (Fig 2w *vs*. 2T). Western blot analysis also showed that the expression of S-Vcanb protein was increased in the Amiodarone-treated embryos, but it was reduced when *s-vcanb* was knocked down (Fig 2G and S2B Fig), indicating that Amiodarone is able to induce *s-vcanb* overexpression, and Amiodarone effects on *cdh5* overexpression is dependent on overexpressive *s-vcanb*, as stated above.

### The EGF motif of S-vcanb is involved in regulating *snail1b* and *cdh5* expression

**Fig 3:**
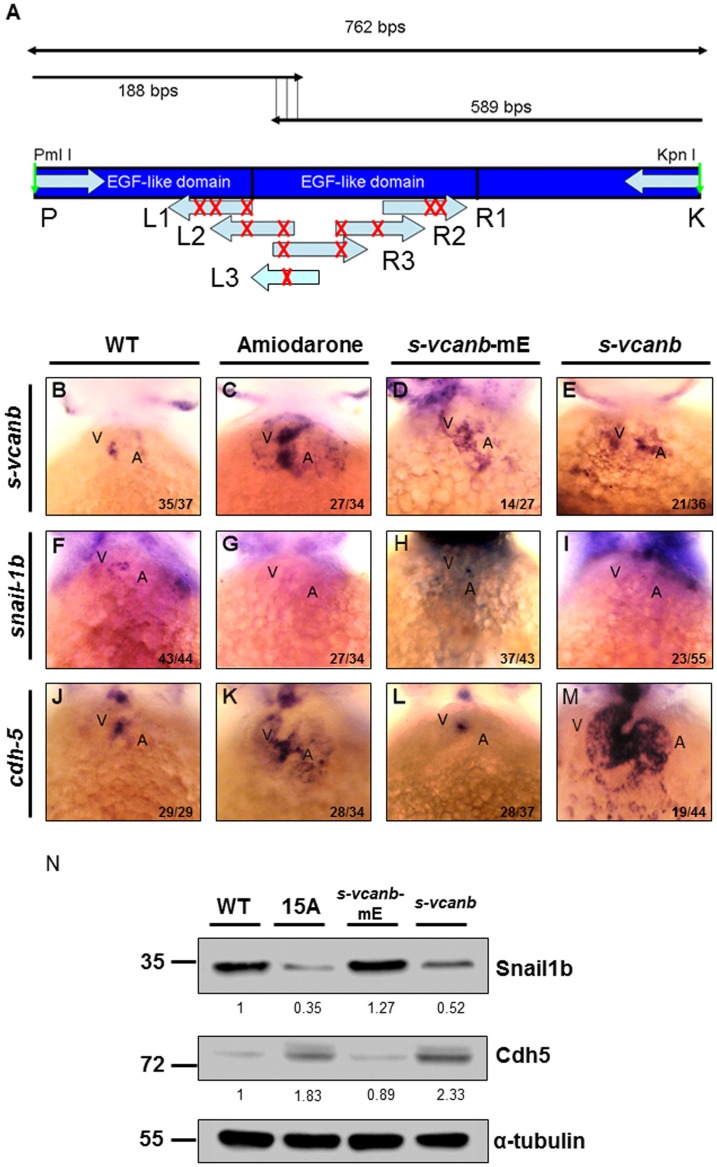
Amiodarone influences S-vcanb/Snail1b/Cdh5 signaling pathway through the EGF motif of S-vcanb. (A) Diagram illustrating the mutation sites of S-vcanb at the EGF motif. Six primers (L1–3 and R1–3) containing eight mutation sites (G→C, indicated with X), which abolish the relativity of EGF motif, were used to generate the pS-vcanb-mE. WISH of *s-vcanb* (B-E), *snail1b* (F-I) and *cdh5* (J-M) in embryos treated with Amiodarone (C, G, K), overexpressed mutation form of *s-vcanb-mE* (D, H, L), or wild-type *s-vcanb* (E, I, M). In Amiodarone-treated embryos, *s-vcanb* (C) and *cdh5* (K) were ectopically expressed, but *snail1b* (G) was lost. In embryos overexpressed in the mutated form of *s-vcanb-mE*, *s-vcanb* (D) was increased, but *snail1b* (H) and *cdh5* (L) levels were similar to those of control group (F and J). In embryos overexpressed with the wild-type form of *s-vcanb*, *s-vcanb* was increased, but *snail1b* was lost, while *cdh5* was greatly ectopically expressed. (N) Western blot analysis of Snail1b and Cdh5 in wild-type embryos, 72 hpf zebrafish embryos treated with 15 μM Amiodarone from 55 to 72 hpf (15 A), overexpressed mutation form of *s-vcanb-mE*, and wild-type *s-vcanb* embryos. Overexpressive wild-type *s-vcanb* embryos displayed reduced Snail1b and increased Cdh5 patterns. The number of embryos displaying a similar pattern was indicated in each figure. The relative intensities of each protein were as indicated. The α-tubulin was used as an internal control.

We employed PCR-based *in vitro* mutagenesis and transgenic assays to test whether the EGF motif of S-Vcanb is involved in Amiodarone-mediated inhibition of zebrafish cardiac valve development. The zebrafish S-Vcanb EGF motif contains eight defined cysteine residues to form specific disulfide bridges responsible for the secondary structure. Following the report of Schrijver et al. [36], we mutated eight cysteine residues into arginine to disrupt the specific disulfide bridges (Fig 3A) and termed the resulting mutated protein as S-Vcanb-mE. Plasmid containing wild-type (WT) S-Vcanb or S-Vcanb-mE, which is driven by CMV promoter, was injected into one-celled embryos and analyzed at 72 hpf. The *s-vcanb* signal was ectopically expressed at the AVC of embryos injected with WT S-Vcanb (Fig 3E *vs*. 3B), indicating that the injected plasmid DNA was transcribed in the cardiac cells. Similar to the pattern shown in the Amiodarone-treated embryos (Fig 3C, 3G and 3K), *snail1b* expression was absent (Fig 3I), while, at the same time, ectopic expression of *cdh5* (Fig 3M) was observed in the *s-vcanb-*overexpressed embryos, suggesting that the effects of Amiodarone on cardiac valve development are S-Vcanb-dependent. At the AVC of embryos injected with mutated S-Vcanb-mE, *s-vcanb* signaling was still ectopically expressed (Fig 3D), and *snail1b* was also expressed (Fig 3H), while *cdh5* expression was limited to the AVC with no ectopic distribution (Fig 3L). Western blot analyses confirmed our WISH data in which the level of Snail1b protein was reduced both in the Amiodarone-treated embryos and *s-vcanb*-overexpressed embryos, while it remained unchanged in the *s-vcanb*-mE-injected embryos. Additionally, the level of Cdh5 protein was increased in both the Amiodarone-treated embryos and *s-vcanb*-overexpressed embryos, while it remained unchanged in the *s-vcanb*-mE-injected embryos (Fig 3N and S2C Fig). Since neither *snail1b* expression nor *cdh5* expression was affected in S-Vcanb-mE-injected embryos, the EGF motif of S-Vcanb is involved in regulating the expressions of Snail1b and Cdh5.

### Role of EGFR signaling

**Fig 4:**
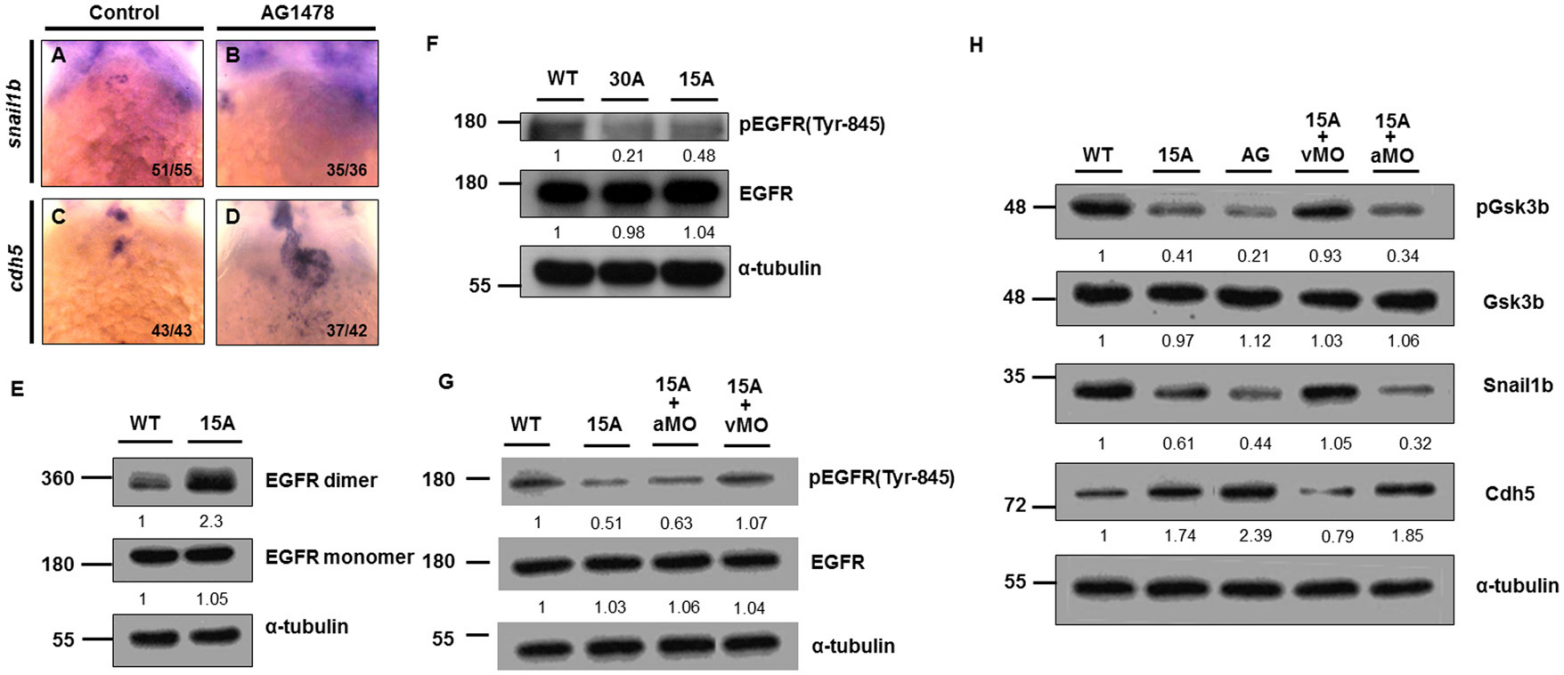
Amiodarone-induced zebrafish *s-vcanb* can repress EGFR signaling. WISH of *snail1b* (A, B) and *cdh5* (C, D) in control (A, C) and AG1478 (B, D)-treated embryos. When AG1478 inhibited EGFR activity, *snail1b* (B) in the AVC was lost, but *cdh5* (D) was greatly induced. The number of embryos displaying a similar pattern was indicated. (E) EGFR-targeting antibodies were used to depict EGFR monomers and dimers in 72 hpf WT and Amiodarone-treated embryos. (F) Amiodarone can repress the phosphorylation of EGFR on Tyr-845. (G) Knockdown of *s-vcanb* (vMO) can rescue the reduction of EGFR phosphorylation on Tyr-845 in Amiodarone-treated embryos, but knockdown of *vcana* (aMO) cannot. (H) Although Gsk3b activity was increased in Amiodarone-treated embryos, it could be rescued by knockdown of *s-vcanb*, but not *vcana*. 15A: 15μM Amiodarone treatment; 30A: 30 μM Amiodarone treatment. The relative intensities of each protein were as indicated. The α-tubulin was used as an internal control.

To prove that EGFR signaling regulates Snail1b and Cdh5 during zebrafish embryogenesis, we treated zebrafish embryos at 55 to 72 hpf with an EGFR inhibitor, AG1478, at a concentration of 7 μM. Results showed that the expression of *snail1b* was absent (Fig 4B *vs*. 4A) and that the expression of *cdh5* was ectopically expressed (Fig 4D *vs*. 4C) at the AVC. These phenotypes were similar to those of Amiodarone-treated embryos and *s-vcanb*-overexpressed embryos. While Amiodarone was able to increase EGFR dimerization (Fig 4E), we noted that the phosphorylation of EGFR was reduced in Amiodarone-treated embryos in a dose-dependent manner (Fig 4F). The pEGFR(Y845) was reduced 79% in embryos treated with 30 uM Amiodarone, while it was reduced 52% in embryos treated with 15 uM Amiodarone. Importantly, knockdown of *s-vcanb* could rescue the reduced EGFR phosphorylation caused by Amiodarone, whereas knockdown of *vcana* could not (Fig 4G and S2D Fig), suggesting that inhibition of EGFR activity by Amiodarone is dependent on *s-vcanb* expression.

**Fig 5:**
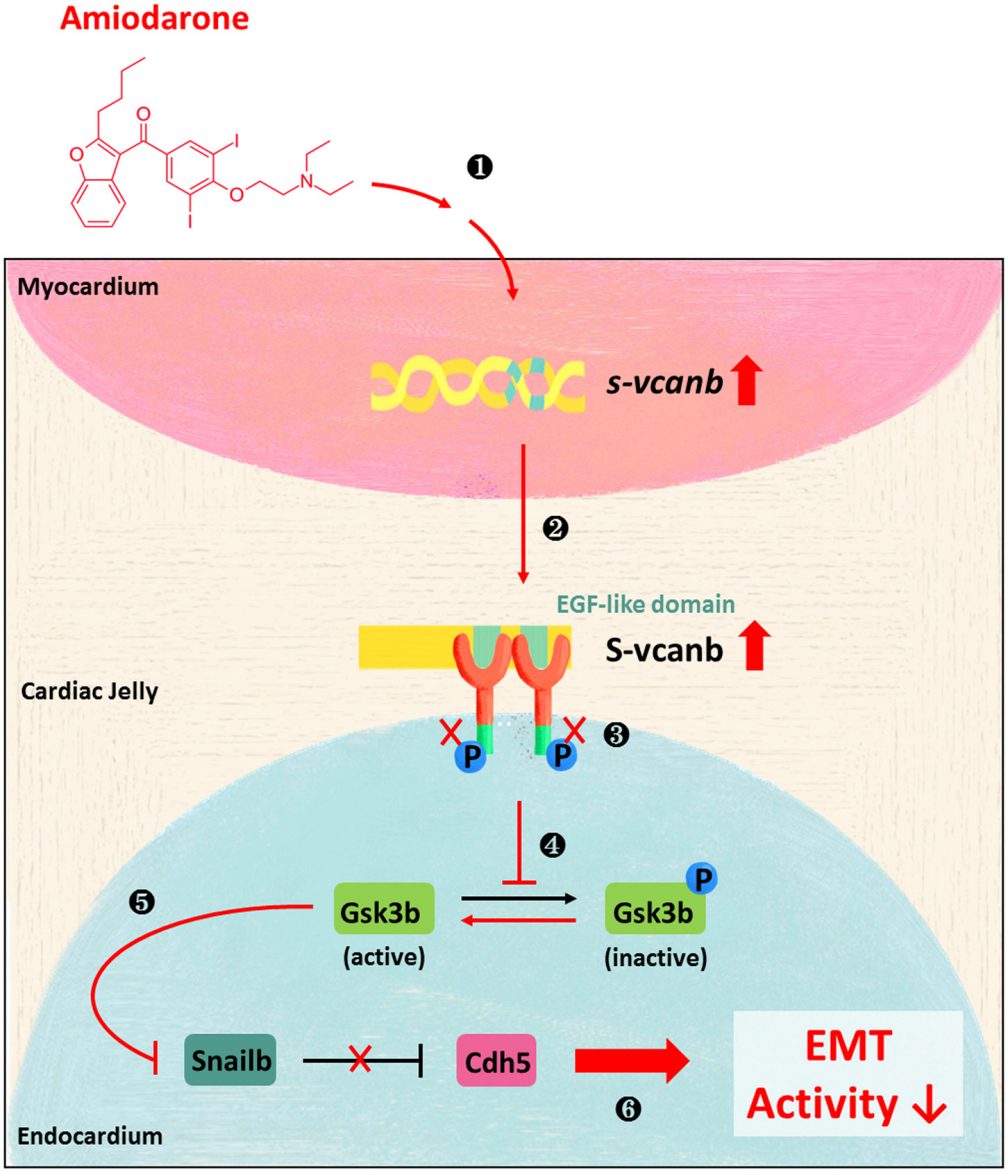
The effect of Amiodarone on cell signaling pathways during cardiac valve formation. (1) Amiodarone induces ectopic transcription of *s-vcanb* in nucleus. (2) S-vcanb protein was overexpressed in ECM. (3) S-vcanb/EGFR interaction inhibits EGFR signaling. (4) Inhibition of EGFR activity increases Gsk3b activity. (5) Activation of Gsk3b reduces the production of Snail protein. (6) Which increases the amount of E-cadherin protein, thus reducing EMT activity in endocardial cells.

To further investigate the molecular mechanism(s) underlying the effects of Amiodarone on EGFR activity, we carried out a detailed analysis of the downstream effectors of EGFR signaling, in particular Gsk3b which can phosphorylate Snail1b, leading to its degradation. While the total protein level of Gsk3b remained unchanged in WT, Amiodarone-treated and EGFR-inhibited embryos, its activity was greatly increased in both Amiodarone-treated and EGFR-inhibited embryos (Fig 4H and S2E Fig). However, knockdown of *s-vcanb* could block the effects of Amiodarone such that Gsk3b activity remained unchanged. In contrast, knockdown of *vcana* caused an increase in Gsk3b activity (Fig 4H and S2E Fig). Reduced EGFR signaling then inhibits Snail functions through increased Gsk3b activity, thereby up-regulating Cdh5 at the AVC. Knockdown of *vcana* in Amiodarone-treated embryos did not rescue the level of Snail1b or Cdh5, whereas knockdown of *s-vcanb* blocked the effects of Amiodarone because the protein levels of Snail1b and Cdh5 were not changed compared to Wt embryos (Fig 4H and S2E Fig). This line of evidence suggests that Amiodarone induces ectopic expression of S-Vcanb, which, in turn, interacts with EGFR by its EGF motif, resulting in reducing EGFR signaling, then inhibits Snail functions through increased Gsk3b activity, finally up-regulating Cdh5 at the AVC (Fig 5).

## Discussion

In this study, we originally found that Amiodarone treatment causes the inhibition of zebrafish cardiac valve formation, hypothesizing that the EMT processes in cardiac endocardium might be repressed. Upon further investigation, we demonstrated that Amiodarone induces ectopic expression of *s-vcanb* at the AVC, resulting in the inhibition of EGFR/Gsk3b/Snail signaling axis and, finally, the increase of Cdh5. We also demonstrated that that *s-vcanb*, but not *vcana*, affects the expression of downstream genes in endocardium. The expression patterns of *snail1b* and *cdh5* remained unchanged in the heart of embryos injected with dominant negative *s-vcanb*, which is absent of EGF-like domain’s function, indicating that *s-vcanb* in myocardial cell affected the expression of *snail1b* and *cdh5* in endocardial cells through EGF-like domain. Furthermore, we found that Amiodarone increases EGFR dimers formation, but decreases EGFR phosphorylation. The phosphorylation of Gsk3b decreases in the Amiodarone-treated embryos, but it did not change in the Amiodarone-treated embryos injected with *s-vcanb*-MO, indicating Amiodarone-mediated Gsk3b activity is *s-vcanb*-dependent. Taken together, our results suggested that Amiodarone represses EGFR/Gsk3b/Snail signaling, which, in turn, up-regulates *cdh5* at the AVC, and causes the defective formation of cardiac valves.

### Each Versican isoform plays a separate and distinct role during cardiac valve development

Versican belongs to a family of hyaluronan aggregating chondroitin sulfate proteoglycans [37, 38], which are highly expressed during endocardial cushion formation [39, 40]. In transgenic mice carrying a homozygous insertional mutation to disrupt the *versican* gene, the endocardial cushion fails to become populated with mesenchymal cells [41]. Since the ability of Versican to influence cellular proliferation has been reported [15, 16, 38, 42, 43], it was thought that Versican acts as a positive effector for the growth of cushion mesenchymal cells derived from EMT. Many studies also demonstrated that different isoforms of Versican could have opposing functions. For example, V1 isoform promotes neurite outgrowth [15], whereas V2 isoform inhibits axonal and neurite growth [14]. This functional “balance” among isoforms was also supported by Sheng et al. [16], who demonstrated that the V1 isoform is able to enhance cell proliferation, while the V2 isoform inhibits cell proliferation. In the present study, we showed, for the first time, that the inhibition of EMT processes of zebrafish cardiac valve formation caused by Amiodarone results from the ectopic expression of *s-vcanb*, a negative VCAN isoform effector which plays an important role during the process where endocardial cushion becomes populated with mesenchymal cells. Collectively, we proposed that the dynamically balanced expressions of two Versican isoforms may provide a suitable extracellular environment for normal proliferation and differentiation of valve primordial cells.

### Zebrafish S-vcanb inhibit EGFR signaling through G3 and CSα domains

It is well known that Versican possesses one chondroitin sulfate (CS)-attachment domain and two globular domains, which are the N-terminal G1 domain and the C-terminal G3 domain. Both V1 and V2 isoforms contain G1 and G3 domains, whereas V1 isoform possesses CSβ and V2 isoform possesses CSα. The G1 domain of Versican can interact with Hyaluronic acid (HA), allowing the cells to regulate cellular responses through the hyaluronan receptors in coordination with CD44 [44]. Then, CD44 associates with the Erbb family and activates the EGFR signaling [45, 46]. The G3 domain contains two EGF motifs which can interact with EGFR [38, 47, 48]. Sheng et al. [16] proved that CSβ domain is responsible for the activation of EGFR signaling; but the CSα domain is responsible for the suppression of EGFR signaling. Therefore, each domain of Versican may have potential to influence EGFR signaling. In this report, we found that repression of EGFR signaling by S-vcanb is dependent on its EGF motif. The function of S-vcanb on repression of EGFR signaling suggests that zebrafish S-vcanb may be functionally conserved to mammalian Versican V2, which also displays negative effect on EGFR activity [16]. Together, we suggested that the repression of EGFR signaling by S-vcanb is dependent on CSα domain of EGF motif. However, the role of G1 domain on zebrafish S-vcanb requires further study.

### Clinical implications

The toxic effect of Amiodarone on cardiac valve development has been well proven in the present study, with the knowledge of its detailed molecular signaling mechanism. Therefore, it can be reasonably concluded that Amiodarone should be avoided in the pregnant women, especially those in the first trimester.

## Supporting Information

S1 Fig Validating specific inhibition of *vcana*-MO used in zebrafish embryos. The *vcana*-MO (12 ng; aMO) combined with its counterpart mRNA (50 pg) was injected into one-celled stage of zebrafish embryos. Uninjected embryos (A), embryos injected with *eGFP* mRNA alone (B), and embryos injected with *eGFP* mRNA plus *vcana*-MO (C), which served as the control group. The aMO-target-eGFP fusion protein was detected at 24 hpf in embryos injected with a aMO-target-eGFP mRNA (D). The aMO-target-eGFP fusion protein was nearly undetectable at 24 hpf in embryos injected with aMO-target-eGFP mRNA plus *vcana*-MO (E).
(DOCX)

S2 Fig Statistical analyses of the densitometric quantification of bands shown on Western blot. The different levels of the intensities of bands shown on Western blot in Figs 1J, 2G, 3N, 4G and 4H were densitometrically quantified and performed statistical analysis using Student’s t-test, which were illustrated in panels A, B, C, D and E, respectively. Data are presented as mean±SD. **P<0.01 and *** P <0.005 indicated the levels of significant difference.
(DOCX)

S1 Table Primer list. (DOCX)
