# Supplementary material for: Amiodarone Induces Overexpression of Similar to Versican b to Repress the EGFR/Gsk3b/Snail Signaling Axis during Cardiac Valve Formation of Zebrafish Embryos
Source: PLoS One. 2015 Dec 9;10(12):e0144751. doi: 10.1371/journal.pone.0144751 (PMC4674151; doi:10.1371/journal.pone.0144751)
Supplement: S1 Fig — The vcana-MO (12 ng; aMO) combined with its counterpart mRNA (50 pg) was injected into one-celled stage of zebrafish embryos. Uninjected embryos (A), embryos injected with eGFP mRNA alone (B), and embryos injected with eGFP mRNA plus vcana-MO (C), which served as the control group. The aMO-target-eGFP fusion protein was detected at 24 hpf in embryos injected with a aMO-target-eGFP mRNA (D). The aMO-target-eGFP fusion protein was nearly undetectable at 24 hpf in embryos injected with aMO-target-eGFP mRNA plus vcana-MO (E). (DOCX) [file pone.0144751.s001.docx]

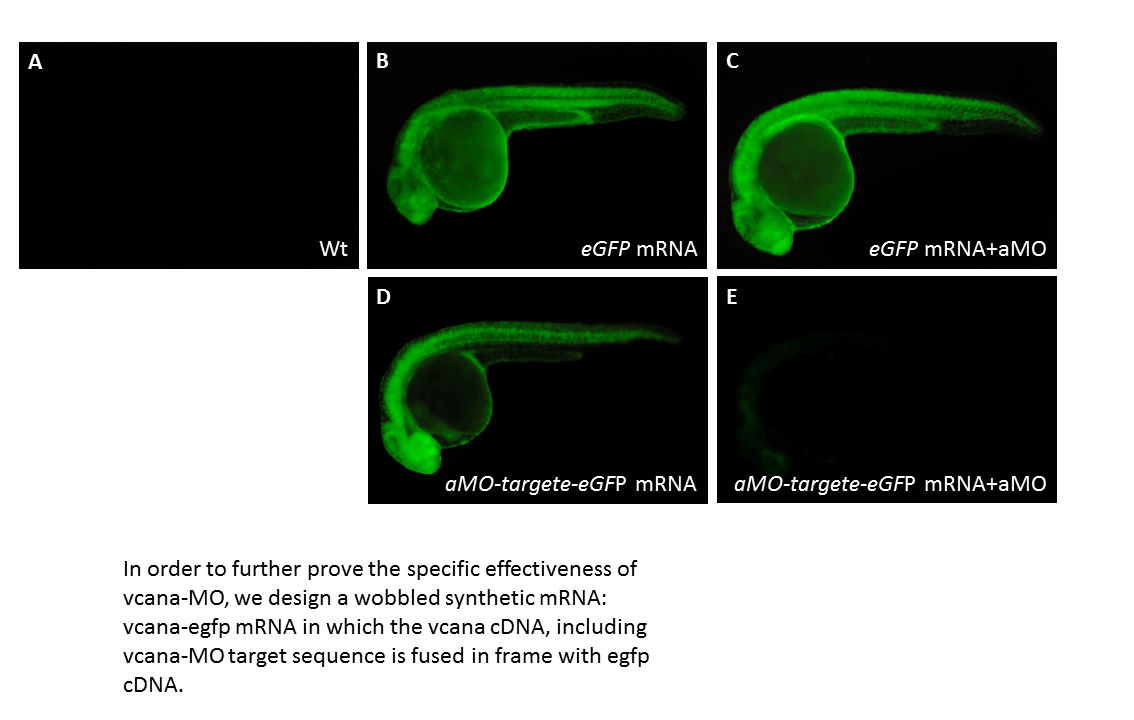


**Figure S1**. Validating specific inhibition of *vcana*-MO used in zebrafish embryos. The *vcana*-MO (12 ng; aMO) combined with its counterpart mRNA (50 pg) was injected into one-celled stage of zebrafish embryos. Uninjected embryos (A), embryos injected with *eGFP* mRNA alone (B), and embryos injected with *eGFP* mRNA plus *vcana*-MO (C), which served as the control group. The aMO-target-eGFP fusion protein was detected at 24 hpf in embryos injected with a aMO-target-eGFP mRNA (D). The aMO-target-eGFP fusion protein was nearly undetectable at 24 hpf in embryos injected with aMO-target-eGFP mRNA plus *vcana*-MO (E).
