# Supplementary material for: Amiodarone Induces Overexpression of Similar to Versican b to Repress the EGFR/Gsk3b/Snail Signaling Axis during Cardiac Valve Formation of Zebrafish Embryos
Source: PLoS One. 2015 Dec 9;10(12):e0144751. doi: 10.1371/journal.pone.0144751 (PMC4674151; doi:10.1371/journal.pone.0144751)
Supplement: S2 Fig — The different levels of the intensities of bands shown on Western blot in Figs 1J, 2G, 3N, 4G and 4H were densitometrically quantified and performed statistical analysis using Student’s t-test, which were illustrated in panels A, B, C, D and E, respectively. Data are presented as mean±SD. **P<0.01 and *** P <0.005 indicated the levels of significant difference. (DOCX) [file pone.0144751.s002.docx]

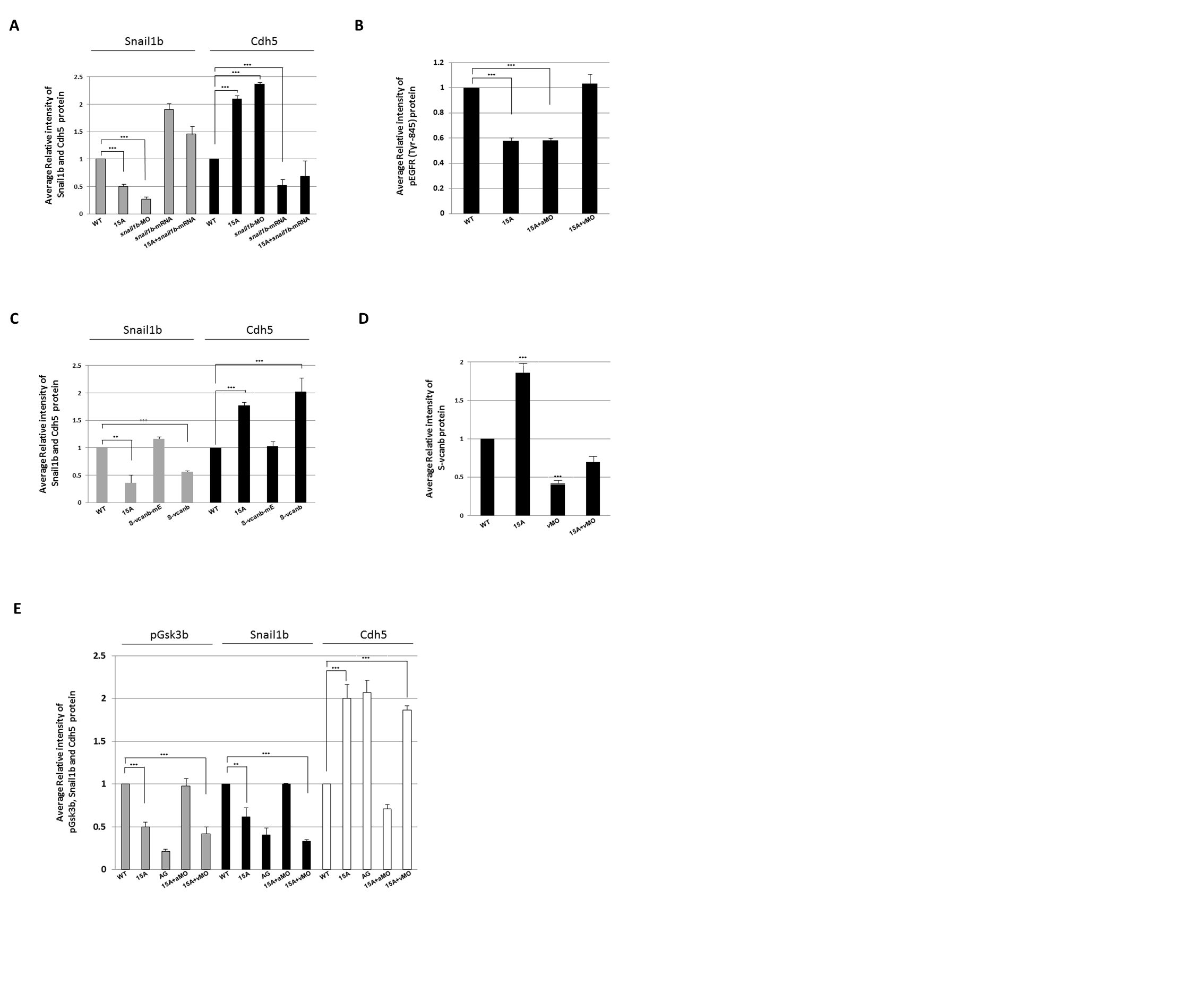
**Figure S2.** Statistical analyses of the densitometric quantification of bands shown on Western blot. The different levels of the intensities of bands shown on Western blot in Figures 1J, 2G, 3N, 4G and 4H were densitometrically quantified and performed statistical analysis using Student’s t-test, which were illustrated in panels A, B, C, D and E, respectively. Data are presented as mean±SD. **P<0.01 and *** P <0.005 indicated the levels of significant difference.
