# Supplementary material for: Amiodarone Induces Overexpression of Similar to Versican b to Repress the EGFR/Gsk3b/Snail Signaling Axis during Cardiac Valve Formation of Zebrafish Embryos
Source: PLoS One. 2015 Dec 9;10(12):e0144751. doi: 10.1371/journal.pone.0144751 (PMC4674151; doi:10.1371/journal.pone.0144751)
Supplement: S1 Table — (DOCX) [file pone.0144751.s003.docx]

| Primer name | Sequences |
| --- | --- |
| Z-vcana-1.0F | AAGGATCCGTGCAGGCCT |
| Z-vcana-2.0R | GGATCGATTTCACTTTAATGATGAT |
| Z-vcana-5UTR-F | ATGTTTTCAGTCCTTTGCGAGTTCGGTGCT |
| Z-vcana-codingR-R | ACTGCCAGAATGACAGTATCCCCAGCATCC |
| Z-vcanb-3.3F | ATTGTCTTTCACCACTGCCC |
| Z-vcanb-3.9R | CACAGCGTTCCCCACTAAAT |
| Z-s-vcanb *Cla*I F1 | ATCGATGTCCACTCCCATGGG |
| Z-s-vcanb *Mfe*I R1 | CAATTGTAAGGGCAAAAGGTGAAGA |
| Z-s-vcanb *MfeI* F2 | CAATTGTTACAGAGGAAGCAGCTG |
| Z-s-vcanb *Hpa*I R2 | GTTGGTTCATATTGGTTTGTTAACTGA |
| Z-s-vcanb *Hpa*I F3 | CAGAAGCTTCAAGTCAGTTAACAAACC |
| Z-s-vcanb *Xba*I R3 | CATTCTCTAGAACAAGTGGTTTAGGGC |
| Z-s-vcanb *Xba*I F4 | CAGCCCACCATTCTCTAGAACAAG |
| Z-s-vcanb *Afe*I R4 | AGCGCTATTGTTTTGTCCGAATGTGT |
| Z-snail1a-F | ATGCCTCGGTCTTTCCTGGTAAAG |
| Z-snail1a-R | CTATTGGACATTGGCCGTGGAGGG |
| Z-snail1b-F | ATCGATATGCCACGCTCATTTC |
| Z-snail1b-R | TCTAGACTAGAGCGCCGGACAGC |
| Z-snail2-F | ATGCCTCGTTCATTCCTAG |
| Z-snail2-R | TCAGTGTGCGATGCAACAG |
| Z-snail3-F | ATGCCAAGGTCTTTCTTG |
| Z-snail3-R | CTAGGACATGGGACAGCA |

Table S1. Primer list
